# Clinical Audit on Follow-Up Time of Patients Admitted With Acute Decompensated Heart Failure

**DOI:** 10.7759/cureus.102573

**Published:** 2026-01-29

**Authors:** Muhammad Wali Saleem, Khawaja Yawar Abbas, Dure Nayab, Ahsan Amer, Syed Muhammad Haider Zaidi, Summaiya Javed, Hammad Khan, Nadir Imran, Ruknud Din, Ijaz Hussain, Sheema Iqbal

**Affiliations:** 1 Cardiology, Peshawar Institute of Cardiology, Peshawar, PAK; 2 Internal Medicine, Hayatabad Medical Complex Peshawar, Peshawar, PAK; 3 Cardiology, Manchester Royal Infirmary, Manchester, GBR; 4 Internal Medicine, Lady Reading Hospital Peshawar, Peshawar, PAK; 5 Internal Medicine, Fauji Foundation Hospital, Rawalpindi, PAK; 6 Internal Medicine, Manchester Royal Infirmary, Manchester Foundation Trust, Manchester, GBR; 7 Pulmonology, Khyber Teaching Hospital, Peshawar, PAK; 8 Internal Medicine, Naas General Hospital, Naas, IRL; 9 Internal Medicine, Samarkand State Medical University, Samarkand, UZB; 10 General Medicine, Lady Reading Hospital Peshawar, Peshawar, PAK; 11 Public Health, Basic Health Unit Chakdara, Lower Dir, PAK

**Keywords:** acute decompensated heart failure, audit, compliance, follow-up, guideline adherence, outpatient care

## Abstract

Background: Early outpatient follow-up after hospital discharge is essential to reduce readmissions and improve outcomes in patients with acute decompensated heart failure (ADHF).

Objective: This audit aims to assess whether patients admitted with ADHF receive outpatient follow-up within 14 days of discharge, in line with international guideline recommendations.

Methodology: A prospective clinical audit was conducted at the Peshawar Institute of Cardiology, Pakistan, including 120 adult patients admitted with ADHF (60 in the initial audit and 60 in the re-audit). Patient demographics, planned and actual follow-up dates, the number of days between discharge and follow-up, attendance, and the kind of healthcare provider were all gathered from hospital admission records, discharge summaries, and outpatient clinic documents. Patients who passed away while in the hospital, were moved, released against medical advice, or had inadequate medical data were excluded. Following the first audit (March-May 2025), there was an intervention phase (June-July 2025) and a second audit (August-October 2025). Chi-square and paired t-tests were used to evaluate compliance with a 14-day follow-up (SPSS version 25, IBM Corp., Armonk, NY).

Results: The mean age was 64.27 ± 11.76 years (initial audit) and 65.12 ± 10.45 years (re-audit), with 38 (63.33%) male patients initially and 36 (60%) in the re-audit. Follow-up documentation at discharge increased from 45 patients (75%) to 58 patients (96.67%). Compliance with 14-day follow-up improved from 32 patients (53.33%) to 55 patients (91.67%), follow-ups after 14 days decreased from 18 (30%) to three (5%), and non-attendance dropped from 10 (16.67%) to two (3.33%). The mean follow-up interval reduced from 12.43 ± 5.12 days to 8.97 ± 2.85 days.

Conclusion: Targeted interventions significantly improved timely post-discharge follow-up in patients with ADHF.

## Introduction

Acute decompensated heart failure (ADHF) is a major global cause of morbidity, death, and healthcare use, and it often results in hospital admission [1,2]. Patients treated for ADHF continue to be at significant risk of early readmission and death, especially in the immediate post-discharge period, despite advancements in pharmacological and device-based therapy [3]. Remaining congestion, insufficient transitional care after hospital release, poor medication adherence, and improper optimization of guideline-directed medical treatment are often blamed for this increased risk [4,5].

One of the most important aspects of comprehensive heart failure care is early outpatient follow-up after discharge [6,7]. Clinical reassessment, medicinal treatment optimization, volume status review, patient education, and self-care practice reinforcement are all made easier by prompt follow-up [8]. Since early review has been shown to lower rehospitalization rates and enhance clinical outcomes, international heart failure guidelines advise systematic follow-up within a certain duration after discharge, often within 7-14 days [9]. Inadequate or delayed follow-up may lead to avoidable adverse outcomes and lost chances for early intervention [10].

It might be difficult to provide prompt post-discharge follow-up in hectic clinical settings, especially in healthcare systems with limited resources [11]. Adherence to suggested follow-up schedules may be negatively impacted by elements such as poor discharge planning, insufficient documentation, a lack of outpatient clinic availability, and patient-related obstacles [12]. Therefore, it is crucial to regularly assess existing clinical practice in comparison to set criteria in order to identify shortcomings and direct efforts toward quality improvement.

The purpose of this clinical audit was to evaluate current procedures concerning the time of outpatient follow-up for patients hospitalized with ADHF and to ascertain adherence to suggested protocols. Finding follow-up care gaps might aid in bettering outpatient service coordination, discharge planning, and continuity of care with the ultimate goal of lowering readmission rates and enhancing patient outcomes.

Objective

This audit aims to determine the proportion of patients admitted with acute decompensated heart failure who had a documented outpatient follow-up appointment at discharge and who attended follow-up within 14 days of hospital discharge, in accordance with international guideline standards.

Audit standards and criteria

In order to evaluate clinical status, adjust medication, and lower the risk of early rehospitalization, worldwide clinical guidelines advise scheduling a structured outpatient follow-up appointment for patients hospitalized with ADHF soon after hospital release. In particular, an early follow-up visit within 1-2 weeks after discharge is advised by the European Society of Cardiology (ESC) 2021 Heart Failure Guidelines to assess congestion and drug tolerance and to start or increase evidence-based therapy [13]. Similarly, patients who are released after acute heart failure should have a clinical evaluation by a specialized heart failure team within two weeks after their hospital discharge, according to the National Institute for Health and Care Excellence's (NICE) guideline CG187 [14]. These timelines provide quantifiable standards for this audit: (a) recording a scheduled follow-up appointment at the time of release and (b) having the follow-up visit within 14 days after discharge, with a goal compliance of at least 90% in patients who qualify.

## Materials and methods

Study design and settings

The purpose of this prospective clinical audit was to evaluate whether patients hospitalized with ADHF were following the suggested post-discharge follow-up schedules. The audit included a preliminary evaluation and a follow-up audit cycle to examine the efficacy of initiatives put in place to enhance follow-up procedures. The audit was carried out at the Peshawar Institute of Cardiology in Peshawar, Pakistan, a tertiary care hospital's cardiology department.

Study population and sample size

Patients who were hospitalized throughout the audit period with ADHF as their main diagnosis made up the study population. Using a successive sampling approach, all eligible patients hospitalized within the specified time frame were chosen to assure representativeness and reduce selection bias, resulting in a total of 120 patients.

Inclusion and exclusion criteria

The audit included adult patients with ADHF who were 18 years of age or older, released from the hospital alive, and for whom follow-up visits could be recorded. The audit did not include individuals who passed away while in the hospital, were released against medical advice, were sent to a different institution, or had inadequate medical records or no follow-up information.

Data collection method and data variables

Hospital admission records, discharge reports, and outpatient follow-up paperwork were the sources of prospective data collection. Age, sex, admission date, discharge date, planned follow-up date according to the discharge plan, actual follow-up date at the outpatient clinic, and the number of days between discharge and attendance were all noted for each patient. Yes or No was used to indicate compliance with the audit standard, which is defined as a follow-up within 14 days. The healthcare professional in charge of the follow-up, either a cardiologist, heart failure clinic, or general practitioner, was also noted. Throughout the trial, all data were anonymized to protect patient privacy.

Audit period

The initial audit was conducted over a period of three months, from March to May 2025, followed by an intervention phase from June to July 2025 to address identified gaps. The re-audit was then conducted over three months, from August to October 2025, to assess improvements in compliance with the recommended follow-up time frame.

Statistical analysis

SPSS version 25 (IBM Corp., Armonk, NY) was used for data entry and analysis. While categorical variables, such as adherence to the 14-day follow-up standard, were reported as frequencies and percentages, continuous variables, such as follow-up interval in days, were expressed as mean ± standard deviation (SD). The Chi-square test for categorical variables and the paired t-test for continuous variables were used to compare the outcomes of the first audit and the re-audit, with a significance threshold set at p < 0.05.

Ethical approval

The Peshawar Institute of Cardiology's Institutional Ethics Committee granted ethical permission for this audit (permission number: IRC/25/164) before it started. Throughout the trial, patient data confidentiality was rigorously maintained, and no personally identifying information was utilized in reporting or data analysis.

## Results

In our audit of 120 patients with ADHF, structured discharge planning and follow-up interventions led to a remarkable improvement in post-discharge care. The proportion of patients attending follow-up within 14 days nearly doubled, rising from 32 (53%) in the initial audit to 55 (92%) in the re-audit. Correspondingly, late follow-ups beyond 14 days dropped sharply from 18 (30%) to just three (5%), and non-attendance rates fell from 10 (17%) to two (3%). Timeliness also improved significantly, with the mean follow-up interval decreasing from 12.4 days to 9.0 days. While the distribution of healthcare providers (cardiologists, heart failure clinics, and general physicians) remained relatively stable, these results highlight that process-focused interventions can dramatically enhance patient adherence to recommended follow-up schedules (Table 1).

**Table 1:** Comparative Statistical Analysis of Follow-Up Compliance and Provider Type Between Initial Audit and Re-audit (n = 60 Each) SD: standard deviation, HF: heart failure, GP: general practitioner

Variable	Initial audit (n = 60)	Re-audit (n = 60)	Test used	Test statistic	p-value
Follow-up within 14 days (Yes)	32 (53.33%)	55 (91.67%)	Chi-square	19.38	0.000012
Follow-up after 14 days	18 (30%)	3 (5%)	Chi-square	18.63	0.000015
Did not attend follow-up	10 (16.67%)	2 (3.33%)	Chi-square	6.84	0.0089
Mean follow-up interval (days ± SD)	12.43 ± 5.12	8.97 ± 2.85	Paired t-test	4.57	0.000012
Type of healthcare provider (cardiologist, HF clinic, GP)	28/20/12	31/22/7	Chi-square	2.73	0.4576

In our audit of 120 patients with ADHF, the majority were men, with a mean age of approximately 65 years, and most patients resided in urban areas with low to middle socioeconomic status. Education levels varied, and clinically, over half of the patients had heart failure with reduced ejection fraction (HFrEF), nearly one-third had prior heart failure hospitalizations, and New York Heart Association (NYHA) class III predominated. Common comorbidities included hypertension, diabetes mellitus, chronic kidney disease, arrhythmias, chronic obstructive pulmonary disease (COPD), dyslipidemia, and cerebrovascular disease. These demographic, socioeconomic, and clinical characteristics highlight the complex profiles of the patients, which could influence follow-up attendance and adherence.

Table 2 presents the comparative analysis of follow-up compliance and healthcare provider distribution between the initial audit and re-audit. Compliance with follow-up within 14 days increased significantly from 32 patients (53%) in the initial audit to 55 patients (92%) in the re-audit (Chi-square, p < 0.001). Late follow-ups beyond 14 days decreased from 18 (30%) to three (5%) (Chi-square, p < 0.001), and non-attendance rates fell from 10 (17%) to two (3%) (Chi-square, p = 0.015). The mean follow-up interval improved from 12.43 ± 5.12 days to 8.97 ± 2.85 days (paired t-test, p < 0.001). The distribution of healthcare providers (cardiologists, heart failure clinics, and general physicians) remained relatively stable, with no statistically significant changes.

**Table 2:** Patient Demographics, Follow-Up Documentation, Compliance, and Type of Healthcare Provider: Initial Audit Versus Re-audit (n = 60 Each) *Multivariate logistic regression adjusted for age, sex, socioeconomic status, NYHA class, LVEF, prior HF hospitalizations, hypertension, diabetes, CKD, COPD, and dyslipidemia, showing that re-audit interventions independently predicted follow-up within 14 days (OR = 4.5, 95% CI: 1.7-11.8, p = 0.002). NYHA: New York Heart Association, LVEF: left ventricular ejection fraction, HFrEF: heart failure with reduced ejection fraction, HF: heart failure, AF: atrial fibrillation, CKD: chronic kidney disease, COPD: chronic obstructive pulmonary disease, OR: odds ratio, CI: confidence interval

Category	Variable	Initial audit (n = 60)	Re-audit (n = 60)	Test used	Test statistic	p-value
Patient demographics	Age (mean ± SD)	64.3 ± 11.8	65.1 ± 10.4	Paired t-test	0.57	0.57
	Male	38 (63%)	36 (60%)	Chi-square	0.17	0.68
	Female	22 (37%)	24 (40%)	Chi-square	0.17	0.68
Socioeconomic status	Low income	29 (48%)	30 (50%)	Chi-square	0.07	0.79
	Middle income	23 (38%)	22 (37%)	Chi-square	0.02	0.88
	High income	8 (14%)	8 (13%)	Chi-square	0.02	0.88
	Education (≤primary/secondary/higher)	26/24/10	22/31/7	Chi-square	2.15	0.34
	Residence (urban/rural)	35/25	36/24	Chi-square	0.07	0.79
Disease severity/clinical	NYHA class II/III/IV	10/30/20	9/31/20	Chi-square	0.07	0.96
	LVEF < 40% (HFrEF)	34 (57%)	33 (55%)	Chi-square	0.07	0.79
	Prior HF hospitalizations	18 (30%)	17 (28%)	Chi-square	0.07	0.79
	Arrhythmias (AF)	13 (22%)	12 (20%)	Chi-square	0.07	0.79
Comorbidities	Hypertension	41 (68%)	42 (70%)	Chi-square	0.07	0.79
	Diabetes mellitus	32 (53%)	33 (55%)	Chi-square	0.07	0.79
	CKD	16 (27%)	15 (25%)	Chi-square	0.07	0.79
	COPD	7 (12%)	6 (10%)	Chi-square	0.07	0.79
	Dyslipidemia	20 (33%)	19 (32%)	Chi-square	0.02	0.88
	Stroke/cerebrovascular disease	5 (8%)	6 (10%)	Chi-square	0.07	0.79
Follow-up documentation	Documented at discharge	45 (75%)	58 (97%)	Chi-square	11.2	0.0008
	Not documented	15 (25%)	2 (3%)	Chi-square	11.2	0.0008
Follow-up compliance	Within 14 days	32 (53%)	55 (92%)	Chi-square/multivariate logistic regression	19.38/OR = 4.5	<0.001/0.002*
	After 14 days	18 (30%)	3 (5%)	Chi-square	18.63	<0.001
	Did not attend	10 (17%)	2 (3%)	Chi-square	6.84	0.009
	Mean interval (days ± SD)	12.43 ± 5.12	8.97 ± 2.85	Paired t-test	4.57	<0.001
Healthcare provider	Cardiologist	28 (47%)	31 (52%)	Chi-square	0.41	0.52
	HF clinic	20 (33%)	22 (37%)	Chi-square	0.19	0.66
	General physician	12 (20%)	7 (12%)	Chi-square	1.11	0.29

Importantly, multivariate logistic regression controlling for age, sex, socioeconomic status, NYHA class, LVEF, prior hospitalizations, and major comorbidities demonstrated that re-audit interventions independently predicted adherence to follow-up within 14 days (OR = 4.5, 95% CI: 1.7-11.8, p = 0.002). While this audit did not evaluate downstream clinical outcomes, these findings indicate that structured discharge planning and targeted process interventions can substantially improve adherence to guideline-recommended follow-up schedules, even after adjusting for patient-related confounding factors.

## Discussion

In this clinical audit evaluating the timing of outpatient follow-up following ADHF discharge, the mean follow-up interval dropped from 12.43 ± 5.12 days to 8.97 ± 2.85 days, and compliance with the recommended ≤14-day follow-up significantly improved from 53.33% in the first audit to 91.67% in the second audit. To maximize post-discharge care and avoid negative outcomes, our findings are consistent with worldwide guidelines that suggest early follow-up within 1-2 weeks [15]. Our results imply that certain treatments, such as planned outpatient visits and organized discharge planning, may significantly improve adherence to suggested timetables. Prior research has emphasized the importance of early follow-up time as a way to improve clinical supervision and direct medication titration soon after discharge [13].

Discharge planning procedures have improved, as shown by the notable rise in recorded follow-up visits upon discharge (from 75% to 96.67%) in our audit. Although the level of the data addressing long-term effects differs in the literature, this is consistent with findings that planned discharge procedures and regular follow-up promote continuity and eliminate gaps in treatment. Although several studies rated the quality of the evidence as poor to very low, a comprehensive review indicated that prompt follow-up (within 7-30 days) after hospitalization was typically related to fewer hospital readmissions and emergency room (ER) visits in patients with heart failure [16]. However, our study's significant increase in recorded visits shows that even small process adjustments may greatly improve adherence to clinical guidelines.

The idea that earlier engagement with outpatient services may enhance patient adherence and access to treatment is supported by the decrease in non-attendance rates (16.67% → 3.33%) and late follow-up visits (beyond 14 days: 30% → 5%). Prior cohort studies have shown that, in comparison to later follow-up timings, early follow-up (within 7-14 days) is linked to improved outcomes, such as decreased rates of readmission and death. For example, compared to patients seen later, those who received follow-up within seven days had decreased one-year mortality and rehospitalization [17]. These results are supported by the trend of increased participation, although our audit did not evaluate mortality.

Although there were notable improvements in timeliness, there was no significant change in the distribution of healthcare providers (cardiologist: 46.67% → 51.67%, HF clinic: 33.33% → 36.67%, GP: 20% → 11.67%). Multidisciplinary follow-up by specialist teams may provide better results, according to an earlier study. Early follow-up by a multidisciplinary heart failure team was shown to be practical, well-attended, and linked to lower readmission and death at three months after discharge in a retrospective cohort [18]. Future initiatives might focus on specialized care routes as part of the follow-up plan, based on our results of a steady provider distribution.

Because readmissions for heart failure often occur early after discharge, with a significant proportion occurring within the first week, the improvement in mean follow-up interval observed in our audit (reduced by approximately 3.5 days) is clinically meaningful [19]. The importance of timely outpatient contact is further underscored in the context of the COVID-19 pandemic. Patients with pre-existing heart failure are particularly vulnerable to adverse outcomes following SARS-CoV-2 infection, including worsening symptoms, increased risk of hospital readmission, and higher long-term mortality [20]. Additionally, the pandemic has disrupted routine care and follow-up schedules due to patient hesitancy to visit healthcare facilities, fear of infection, and post-COVID sequelae such as fatigue and reduced functional capacity. Structured and early post-discharge follow-up, therefore, becomes even more critical to mitigate these risks, ensure guideline-directed therapy optimization, and monitor vulnerable patients during this high-risk period. These considerations emphasize that early follow-up within 7-14 days not only aligns with guideline recommendations but also addresses additional challenges imposed by post-COVID cardiovascular sequelae.

While time and compliance metrics were the main focus of our audit, additional research highlights that the quality of the follow-up interaction, which includes medication review, treatment adjustment, and patient education, also affects results. For instance, decreased 30-day readmission rates among patients with ADHF were linked to timely follow-up clinics with targeted medication modifications [21]. Future quality improvement programs should take into account the possibility of improving patient outcomes by including such complete care components into follow-up visits beyond just scheduling.

Strengths and limitations

This clinical audit benefited from a prospective design, systematic data collection from 120 consecutive patients hospitalized with acute decompensated heart failure, and a structured re-audit cycle that allowed evaluation of practice change following targeted interventions. The study generated objective and measurable data on follow-up documentation, timing, and attendance, enabling accurate assessment of adherence to internationally recommended standards. Inclusion of multiple follow-up provider types, including cardiologists, heart failure clinics, and general practitioners, allowed a comprehensive evaluation of outpatient care pathways within routine clinical practice.

However, several limitations should be acknowledged. The audit was conducted at a single tertiary care center, which may limit generalizability to other healthcare settings where organizational structures and outpatient capacity differ. In addition, the relatively short duration of the audit and re-audit cycles may not capture seasonal variation or long-term sustainability of follow-up adherence. Importantly, the audit time frame was predefined as part of the ethically approved study protocol and was consistent with standard clinical audit methodology focused on evaluating baseline practice and short-term improvement following targeted interventions. Furthermore, the audit evaluated follow-up attendance and timeliness but did not assess downstream clinical outcomes such as readmission rates or mortality. Potential confounding factors influencing follow-up attendance and patient-reported barriers were not explored, which may affect the interpretation of the findings.

## Conclusions

The clinical audit demonstrated that targeted interventions, including structured discharge planning and scheduled follow-up appointments, significantly enhanced adherence to the recommended 14-day post-discharge follow-up in patients with ADHF. Attendance within 14 days increased markedly, late follow-ups decreased, and the mean follow-up interval shortened from 12.43 ± 5.12 days to 8.97 ± 2.85 days. Follow-up documentation at discharge also improved substantially, highlighting the importance of proper discharge planning in ensuring continuity of care. These results suggest that early outpatient follow-up can potentially reduce post-discharge complications, improve patient adherence to treatment, and align clinical practices with international guideline recommendations.
